# Isoform-Specific Modulation of Inflammation Induced by Adenoviral Mediated Delivery of Platelet-Derived Growth Factors in the Adult Mouse Heart

**DOI:** 10.1371/journal.pone.0160930

**Published:** 2016-08-11

**Authors:** Radiosa Gallini, Jenni Huusko, Seppo Ylä-Herttuala, Christer Betsholtz, Johanna Andrae

**Affiliations:** 1 Department of Immunology, Genetics and Pathology, Rudbeck Laboratory, Uppsala University, Uppsala, Sweden; 2 Department of Medical Biochemistry and Biophysics, Karolinska Institute, Stockholm, Sweden; 3 Department of Biotechnology and Molecular Medicine, AI Virtanen Institute for Molecular Sciences, University of Kuopio, Kuopio, Finland; Leiden University Medical Center, NETHERLANDS

## Abstract

Platelet-derived growth factors (PDGFs) are key regulators of mesenchymal cells in vertebrate development. To what extent PDGFs also exert beneficial homeostatic or reparative roles in adult organs, as opposed to adverse fibrogenic responses in pathology, are unclear. PDGF signaling plays critical roles during heart development, during which forced overexpression of PDGFs induces detrimental cardiac fibrosis; other studies have implicated PDGF signaling in post-infarct myocardial repair. Different PDGFs may exert different effects mediated through the two PDGF receptors (PDGFRα and PDGFRβ) in different cell types. Here, we assessed responses induced by five known PDGF isoforms in the adult mouse heart in the context of adenovirus vector-mediated inflammation. Our results show that different PDGFs have different, in some cases even opposing, effects. Strikingly, whereas the major PDGFRα agonists (PDGF-A and -C) decreased the amount of scar tissue and increased the numbers of PDGFRα-positive fibroblasts, PDGFRβ agonists either induced large scars with extensive inflammation (PDGF-B) or dampened the adenovirus-induced inflammation and produced a small and dense scar (PDGF-D). These results provide evidence for PDGF isoform-specific inflammation-modulating functions that may have therapeutic implications. They also illustrate a surprising complexity in the PDGF-mediated pathophysiological responses.

## Introduction

Platelet-derived growth factors (PDGFs) are dimeric disulfide-bound growth factors that affect the proliferation, differentiation, migration and survival of various types of mesenchymal cells (reviewed by [1]). The PDGF family consists of four genes (PDGF-A, -B, -C and -D), and their products form 5 biologically active dimeric configurations (AA, BB, AB, CC and DD). Additionally, through alternative splicing, the PDGF-A transcript generates two different protein isoforms: PDGF-A_long_, which binds heparin sulfate proteoglycans in the extracellular matrix, and PDGF-A_short_, which is freely diffusible. Accordingly, PDGF-AA and -AB may occur in further sub-isoforms. PDGFs exert their activities by binding and activating two tyrosine kinase receptors (PDGFRα and -β). Phosphorylation of specific intracellular tyrosine residues of the receptors initiates signaling cascades that control cell growth and division, actin cytoskeleton rearrangements and cell migration. Affinity studies *in vitro* have revealed high-affinity interactions between PDGFRα and –β and different hetero- and homodimers of PDGFs. Despite broad ligand-receptor specificity, only few of the known possible interactions have been linked to physiological roles *in vivo*; these are between PDGFRα and PDGF-AA and -CC, and between PDGFRβ and PDGF-BB, respectively (reviewed in [1]).

The PDGF genes are expressed in many organs and tissues during both development and in adulthood. PDGF-A and -C expression is generally observed in various developing epithelia, whereas PDGFRα is found in the adjacent mesenchymal cells, suggesting a paracrine mode of signaling and a role in epithelial-mesenchymal interaction. An analogous paracrine scenario has been demonstrated for PDGF-B and PDGFRβ. Vascular endothelial cells express PDGF-B, while PDGFRβ is expressed in neighboring vascular mural cells (pericytes and vascular smooth muscle cells) (reviewed in [2]).

PDGF-A and -C expression is commonly observed in cardiac myocytes [3,4], whereas PDGFRα expression is found in neighboring mesenchymal cells/fibroblasts. Cardiac expression of PDGFRα onsets in epicardial cells originating from the secondary heart field. These cells subsequently migrate into the heart as myocardial interstitial cells, coinciding with the onset of myocardial PDGF-A and PDGF-C expression [5]. Genetic loss of PDGFRα signaling leads to failure of formation of epicardium-derived interstitial cells, an overall reduction of fibroblasts and a net decrease in extracellular matrix [6]. Cardiac PDGF-B and PDGFRβ is mainly observed in vascular endothelial and mural cells, respectively, throughout the developing coronary system. Genetic loss-of-function of PDGF-B or PDGFRβ leads to impaired coronary arteriogenesis, septal defects, reduced development of the atrioventricular cushions and valves, and hypoplasia of the myocardium and cardiac nerves, all indicative of an essential role for PDGF-B/PDGFRβ signaling in coronary maturation [7,8].

In addition to developmental roles, PDGFs are implicated in injury-driven inflammation of tissues, such as in a wound healing following endothelial damage. During the initial coagulation response, platelets get activated when exposed to the subendothelial layers of collagens and von Willebrand factor. Activated platelets release growth factors, such as PDGFs and TGF-beta, which attract inflammatory cells and stimulate the activation of local fibroblasts. These, in turn, acquire reactive myofibroblast properties and initiate extracellular matrix synthesis. It is well established that PDGFs influence fibroblast migration, adhesion and extracellular matrix deposition and remodeling [9].

Although reversible collagen deposition is indispensable for wound healing in normal tissue repair, the coagulation cascade may result in excessive accumulation of fibrous connective tissue. That usually happens in conjunction with severe or repetitive injuries or upon chronic inflammation. Permanent scarring often impairs the function of the affected organ, and may even be a deadly condition. Myocardial fibrosis is the terminal stage of several progressive cardiovascular diseases. For example; chronic hypertension may cause progressive interstitial fibrosis and left-ventricular hypertrophy, during which excessive accumulation of extracellular matrix leads to myocardial stiffness and ventricular dysfunction. Activated fibroblasts are considered the main actors during fibrotic heart diseases, but other cell types, such as monocytes/macrophages, lymphocytes, mast cells, vascular cells, as well as the cardiomyocytes themselves, may contribute as sources of pro-fibrogenic mediators (reviewed by [10]). Transgenic overexpression experiments suggest that PDGFs are capable of promoting cardiac fibrosis during development. Different PDGF isoforms have different effects in this regard, with PDGF-A and -C being more fibrogenic than PDGF-B and -D, implicating that signaling via PDGFRα is important [11,12] (Gallini et al, submitted).

It is still unclear whether the ligand-specific developmental effects of PDGFs translate into similarly ligand-specific fibrogenic roles in the adult heart. In this study, we assessed the effects of different PDGF isoforms delivered by adenovirus injections directly into the adult heart. This mode of PDGF expression generates a condition that differs from transgenic expression, since it occurs in the context of a local inflammatory response induced by the adenovirus itself. Indeed, our results reveal surprising differences in the PDGF-elicited effects during inflammation, including PDGF isoform-specific inhibition of the adenovirus-induced inflammatory response, and opposing results exerted by PDGF ligands known to signal through the same receptor. These results were not predicted based on previous knowledge and challenge our views of PDGF biology in disease versus development.

## Materials and Methods

### Ethics statement

This study was carried out in strict accordance with applicable standards. The mice were kept in The National Laboratory Animal Center of the University of Eastern Finland, in standard housing conditions. Diet and water were provided ad libitum. All animal procedures were approved by The National Animal Experiment Board of Finland (permit number ESLH-2008-07516/Ym-23), and carried out in accordance with the guidelines of The Finnish Act on Animal Experimentation. The investigation conforms to the *Guide for the Care and Use of Laboratory Animals* published by the US National Institutes of Health (NIH Publication No. 85–23, revised 1996). All efforts were made to minimize animal suffering.

### Virus-injected animals

Adenoviruses were injected into *Pdgfra*^*GFP/+*^ knock-in mice in C57BL/6J background [13], where one of the *Pdgfra* alleles had been replaced by a H2B-GFP construct. In total, 56 male mice at the age of 9 to 12 weeks were used. Each virus was injected in at least eight mice.

### Viral constructs

Human serotype 5 adenoviruses were produced under GMP conditions in *293* cells, verified to be free from endotoxin and microbiological contaminants. The viruses were replication deficient due to deletion of E1 and partially E3. Adenoviral constructs expressing all different isoforms of PDGFs were generated from cDNAs; 1309bp PDGF-A_long_ (clone D1) [14]; 1240bp PDGF-A_short_ (clone 13.1) [15]; PDGF-B [16]; PDGF-C and PDGF-D (activated form without CUB domain). An empty virus, containing only the CMV promoter (AdCMV), was used as control. All viral constructs were tested in vitro with western blot for protein production of correct size. Viral constructs were diluted in 0.9% saline (www.bbraun.com) to a final concentration of 1x10^12^ viral particles (vp) per milliliter, and a total of 1x10^10^ vp in 10 μl was injected into the myocardium of each mouse.

### Echocardiography-guided myocardial injections

Adenoviral constructs were injected into the anterior wall of the left ventricle under ultrasound guidance. A high-resolution imaging system, specially developed for small animal research (Vevo 770, www.visualsonics.com), was used for echocardiography. The injection procedure has been described before [17]. All mice received analgesic treatment after the operation (carprofen 50 mg/ml, Rimadyl, www.pfizer.com). Baseline echocardiographic measurements were done immediately before the injections, after which the mice were blindly randomized into different treatment groups. Statistical analyses were performed with Student´s t-test in comparison to the corresponding group´s baseline measurements. Echocardiography data was obtained on the injection day (D0) and after 14 days (D14) days, before the mice were sacrificed. A high-frequency ultrasound probe (RMV-707B) operating at 30 MHz, with a focal depth of 12.7 mm was used. The animals were anesthetized with isoflurane (induction: 4.5% isoflurane, 450 ml air, maintenance: 2.0% isoflurane, 200 ml air, www.baxter.com). The mice were placed in supine position on a heated platform (THM100, www.indusinstruments.com) to maintain the body temperature at 36–37°C. The temperature was monitored via a rectal probe, throughout the study protocol. Hair was removed from the chest with a depilatory cream (Veet, www.rb.com) and warm ultrasound gel (Parker Aquasonic 100, www.scidi.com) was applied to the shaved chest. Ejection fraction and left ventricle diastolic wall thickness were determined from short axis M-Mode measurements. Ejection fraction was calculated by Vevo770 software by using the following formula:

$$100\times \frac{LVvol;d-LVvol;s}{LVvol;d}$$

in which $LVvol;d=\frac{7.0}{2.4+LVID;d}\times LVID;d^{3}$

and $LVvol;s=\frac{7.0}{2.4+LVID;s}\times LVID;s^{3}$

in which *LVID;d* = Left ventricular internal diameter (diastole)

and *LVID;s* = Left ventricular internal diameter (systole).

### Fixation and staining of heart tissue

Two weeks after injection, mice were perfusion fixed through the heart with 1% paraformaldehyde (PFA), cut horizontally into halves, postfixed in 4% PFA for 4 hours at 4°C and washed with PBS. Hearts were soaked in a sucrose gradient (10%– 30%) in PBS before embedding in OCT. Cryo sections (25–30 μm) on poly-L-lysine-coated slides were post fixed in 4% PFA for 10 minutes at RT. The sections were permeabilized and blocked in 0.5% Triton-x100; 0.1% BSA in PBS. All antibodies were diluted in 0.1% BSA. Primary antibodies used: rat-anti-mouse CD31, 1.25 μg/ml (553370, www.bdbiosciences.com); Cy3-conjugated mouse-anti-human α-SMA, 2.8 μg/ml (C6198, www.sigmaaldrich.com); anti-mouse PDGFRβ, 2.5 μg/ml (14-1402-82, www.ebioscience.com); rabbit-anti NG2, 10 μg/ml (AB5320, www.millipore.com); PINP, 7.93 μg/ml (Bode et al., Eur J Vasc Endovasc Surg 2002); goat-anti Nkx2.5, 10 μg/ml (AF2444, www.rndsystems.com); rabbit-anti-mouse Ki-67, 3 μg/ml (ab15580, www.abcam.com); rat-anti CD45, 0.125 μg/ml (610266, www.bdbiosciences.com). Secondary antibodies used: Alexa Fluor 555 goat-anti-mouse IgG, 5 μg/ml (A-21424); Alexa Fluor 647 goat-anti-rat IgG, 2 μg/ml (A-21247); Alexa Fluor 647 donkey-anti-goat IgG, 4 μg/ml (A-21447); Alexa Fluor 405 goat-anti-rabbit IgG, 10 μg/ml (A-31556), all from www.invitrogen.com. DyLight conjugated donkey-anti-goat, 3.75 μg/ml (705-475-147); Cy3 conjugated goat-anti-rabbit IgG, 1.5 μg/ml (111-165-144); Cy3 conjugated goat-anti-rat IgG, 7.5 μg/ml (112-165-167), all from www.jacksonimmuno.com. The sections were counter stained with 5 μg/ml FM 4–64 FX membrane dye (F34653, www.invitrogen.com) and imaged using confocal microscopy (Zeiss 700 ZEN). Maximal projections were created from 3 single optical sections acquired with depth 0.684 μm each.

Hematoxylin/Eosin staining was performed on cryo sections (25–30μm) according to routine protocol. Bright field images were obtained using a Zeiss AxioImager microscope. Single images were stitched together to generate over view images using Zen blue software.

### Analysis and statistics of scar tissue

The average scar size in each group was calculated from the scar volume of every sample, with an approximation to the ellipsoid volume with the formula

$$volume=\frac{4}{3}\pi abc$$

where *a*, *b* and *c* are the ellipsoid’s semi-principal axes of length. The three principal axes were obtained by multiplying the thickness of every cryo section with the number of sections containing scar tissue, and measuring the diameters of the widest scar sections using ZEN software (www.zeiss.com). Quantification of PDGFRα positive cells was performed manually and blinded by three persons (RG, CB, JA) directly under the microscope. The average distribution of PDGFRα positive cells was estimated by dividing the number of GFP positive cells by the area of the scar, performed in Volocity 5^®^, Improvision (www.perkinelmer.com).

Results are presented as means ± standard deviation, the statistical significance was evaluated with a paired Student’s t-test, were P < 0.05 was considered statistically significant. The following symbols are used in the figures: * is p < 0.05, ** is p < 0.01 and *** is p < 0.001.

One-way-ANOVA analysis was performed in Prism 6, with correction of Tukey´s multiple comparisons test.

## Results

### Adenovirus injection results in myocardial fibrosis

**Fig 1:**
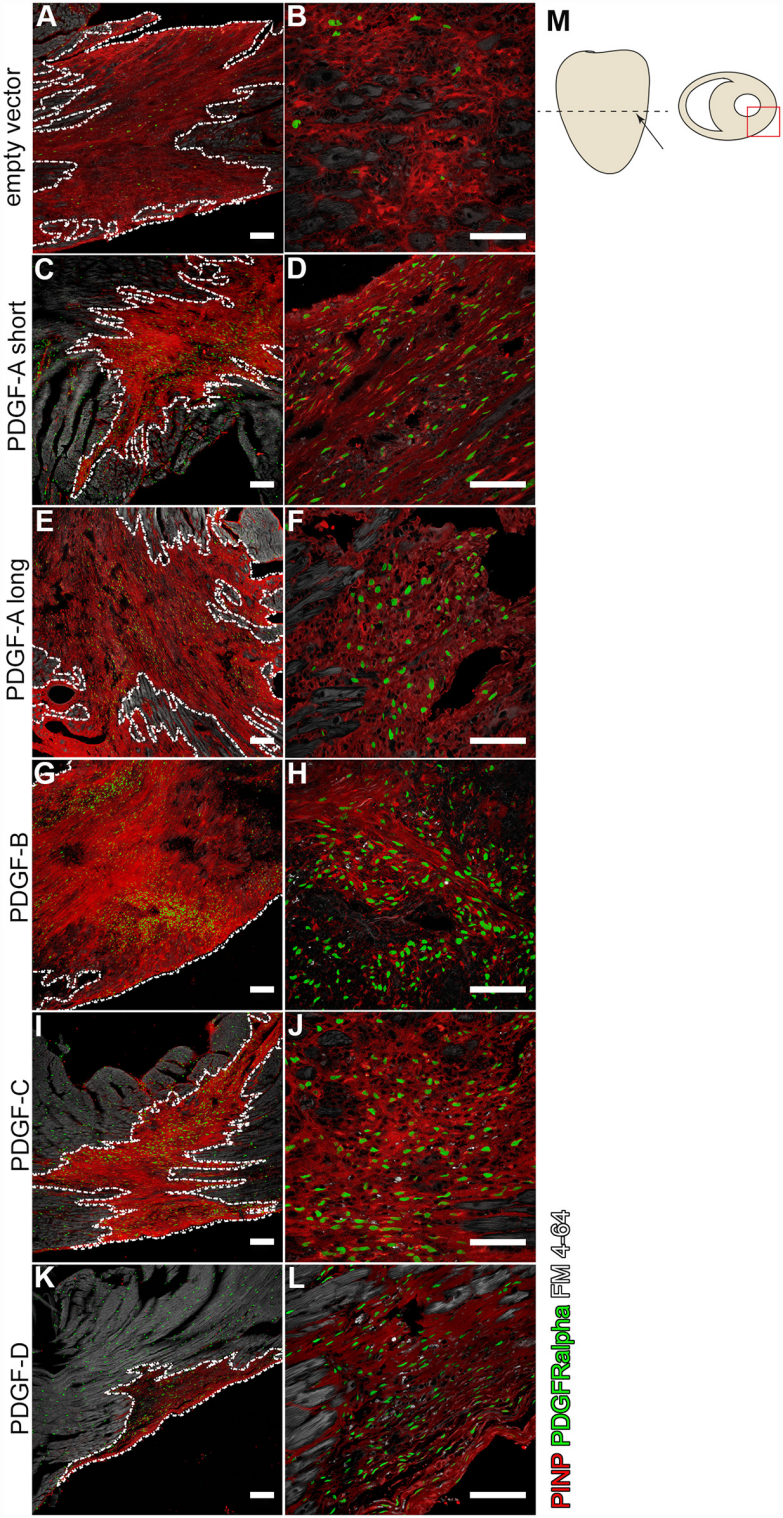
Distribution of the fibrotic scars induced in the myocardium after viral injections. Pro-collagen 1 (PINP) expression (in red) marks the area of the scar. PDGFRα positive nuclei are shown in green. (Left column; A, C, E, G, I, K) The scar size varies in-between the different viruses. White dotted lines outline the perimeter of the scar. (Right column; B, D, F, H, J, L) Green PDGFRα positive cells are increased in all PDGF-virus induced scars. Scale bars are 100 μm. (M) Schematic drawing of a heart illustrating the injection site in the left ventricle wall (arrow). The dotted line shows the section orientation, and the red square show where pictures of scars are taken.

Transgenic overexpression of single PDGFs in the developing heart induces fibrosis with ligand-specific differences [11,12] (Gallini et al, submitted). In this study, we aimed to compare the fibrogenic properties generated by all different PDGF ligand isoforms except the known heterodimers PDGF-AB and PDGF-A_long_A_short_. Adenoviral constructs containing cDNA sequences for *Pdgfa*_short_, *Pdgfa*_long_, *Pdgfb*, *Pdgfc* or *Pdgfd* were injected into the anterior wall of the left ventricle of the heart in adult mice. Empty adenovirus vector was used as control. A minimum of 8 mice was injected for each type of virus. All viruses were injected into *Pdgfra*^*GFP/+*^ knock-in mice, a reporter mouse line that expresses green fluorescent protein (GFP) in PDGFRα positive cells, enabling localization of PDGFRα expressing cells and evaluation of their role in the fibrotic response. Two weeks after injection, tissue damage response (hereinafter referred to as a scar) was observed in the myocardium of all mice. This included the empty adenovirus-injected mice, which developed a significant size local reaction (Fig 1A), on top of which PDGF ligand-specific effects were analyzed.

### Scar size

**Fig 2:**
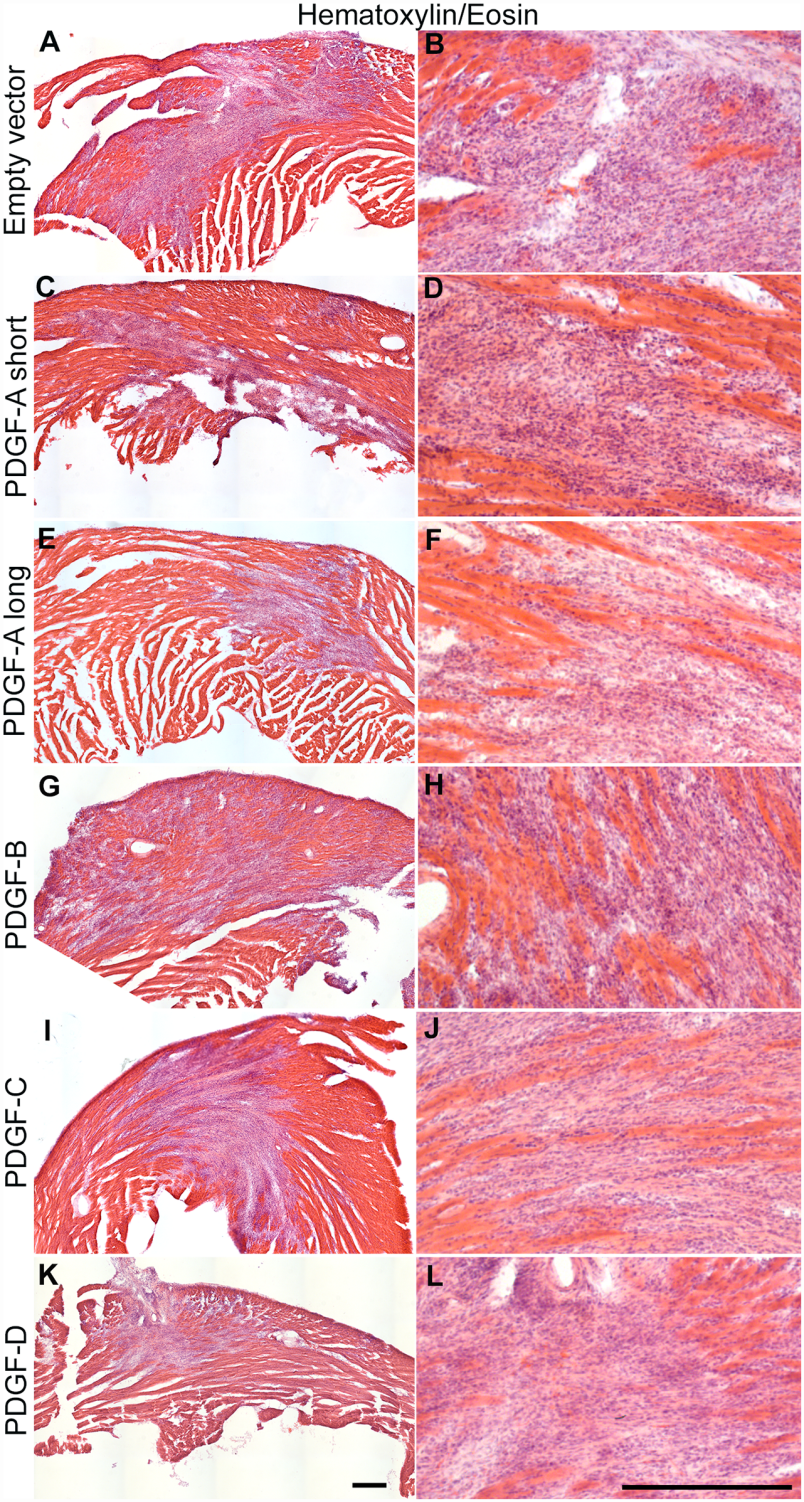
Hematoxylin/eosin staining of virus induced scars generated by different PDGF isoforms. Hematoxylin/eosin staining of cryo sectioned hearts: (A, B) empty vector control; (C, D) PDGF-A_short_ virus; (E, F) PDGF-A_long_ virus; (G, H) PDGF-B virus; (I, J) PDGF-C virus; (K, L) PDGF-D virus. Scale bar is 250 μm.

**Fig 3:**
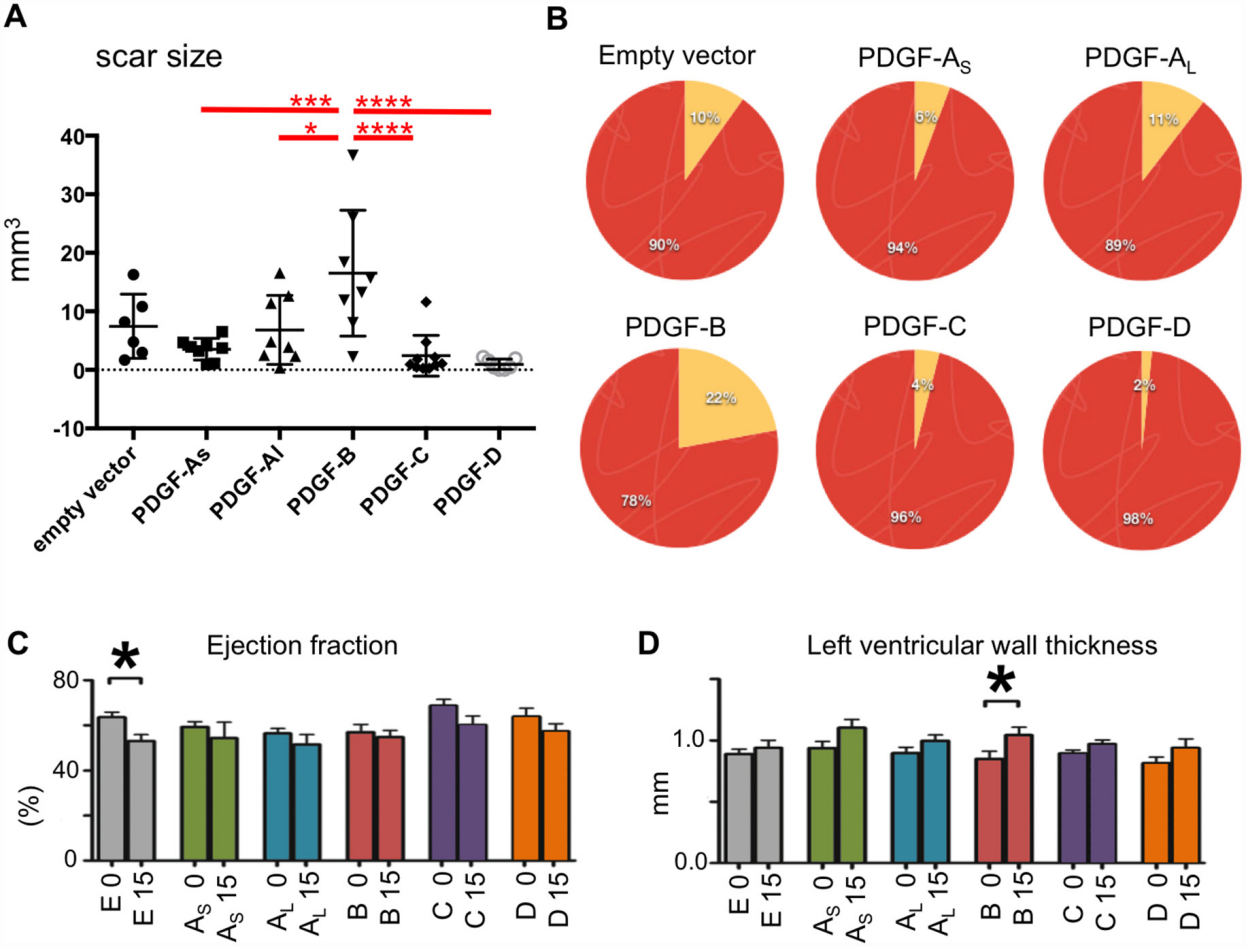
Comparison of scar size and heart functionality. (A) One-way ANOVA analysis of scar volume corrected with Tukey´s multiple comparisons test. (B) Circle diagram illustrating the average volume of scar tissue (yellow) relative to the volume of cardiac tissue (red) for each different type of virus. (C) Ejection fraction of the left ventricle (indicating the ventricular functionality) before, and 15 days after, virus injections. (D) Left ventricle wall thickness before, and 15 days after virus injection. Error bars in D, E show std.dev and asterisk indicate a statistical significant difference (p<0.05). E = empty vector; As = PDGF-A_short_; Al = PDGF-A_long_; B = PDGF-B; C = PDGF-C; D = PDGF-D.

All hearts were serially sectioned through the scars. Scar areas were identified by immunofluorescence staining of collagen producing cells (Fig 1). There were noticeable variances in scar size caused by the different viruses (Fig 1, dotted lines in left column). In general, the scar shapes approximated that of ellipsoids with ragged borders to the surrounding non-fibrotic heart tissue. The scars were also visible by standard histological staining, such as hematoxylin and eosin (Fig 2). The average scar volume for each group was measured and calculated (Fig 3A), and related to the size of the hearts (Fig 3B). Hearts injected with viruses expressing PDGF-B displayed significantly larger scars than hearts injected with the empty vector virus. Surprisingly, PDGF-A_short_, PDGF-C and PDGF-D virus generated scars that were smaller than the empty vector controls. The smallest scars were observed in PDGF-D virus injected hearts, whereas the size of PDGF-A_long_ scars was similar to the size of the empty vector controls.

There was a substantial variability in size between scars in the same experimental group of animals, however, this was in concordance with the expected inter-individual variability in adenoviral-mediated inflammatory response *in vivo* [17,18]. However, analyses of the probability distribution of the scar sizes showed a narrow variability for some of the viruses (Fig 3A). The range in size variation for PDGF-D-induced scars was very low; these scars were substantially smaller than in any of the other mice. The largest variability was observed in scars generated by the PDGF-B virus and the empty vector virus. The asymmetric shape of some of the size distributions might indicate the presence of outliers (see e.g. the PDGF-C scars).

### Ventricular functionality

Heart functionality was evaluated by echocardiography. Left ventricular wall thickness and ejection fraction were measured before and two weeks after virus injection (at the experimental end point). Although there was a tendency towards decreased ejection fraction in all groups, the only significant decrease was observed in the empty vector virus group (Fig 3C). Similar results have been obtained in previous studies [17,18]. Similarly, there was a tendency towards increased left ventricular wall thickness after two weeks in all groups, but the only significant difference was observed in the group injected with PDGF-B virus (Fig 3D). These results were consistent with the large size scars observed in the PDGF-B group.

### PDGFRα positive cells

**Fig 4:**
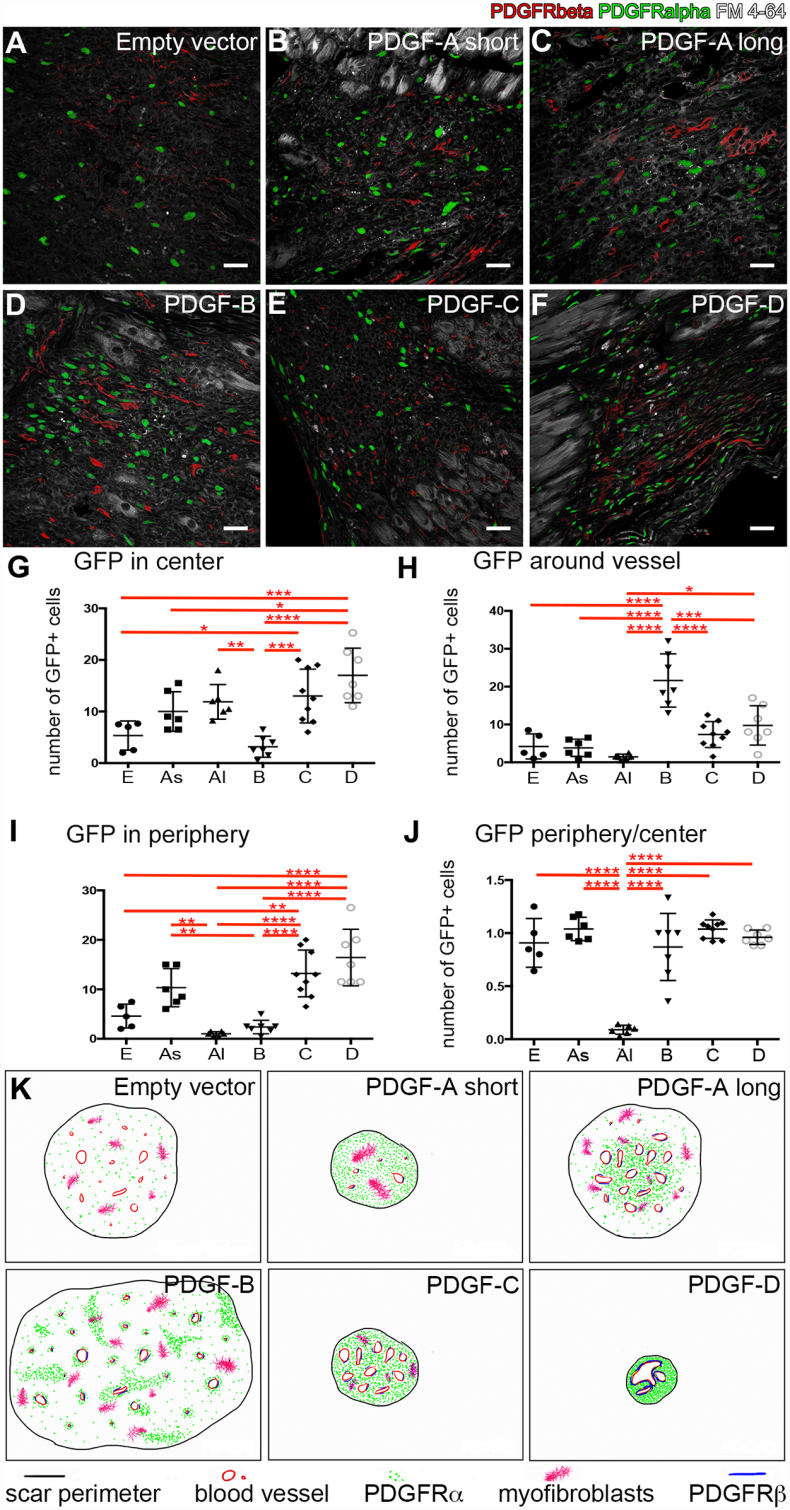
Distribution of PDGF receptors. (A-F) Expression of PDGFRα (green nuclei) and PDGFRβ (red) in cells within the fibrotic scars. Lipid membranes are highlighted in grey. Scale bars are 30μm. (G-J) One-way ANOVA analyses of GFP expression in different locations of the scar, corrected with Tukey´s multiple comparisons test. * = p<0.05; ** = p<0.01; *** = p<0.001, **** = p<0.0001. (G) PDGFRα positive cells in the center (non-peripheral area) of the scar. (H) PDGFRα positive cells in close association to vessels. (I) PDGFRα positive cells in peripheral regions. (J) Relation of PDGFRα positive cells in the peripheral- and central region of the scar. E = empty vector; As = PDGF-A_short_; Al = PDGF-A_long_; B = PDGF-B; C = PDGF-C; D = PDGF-D. (K) Schematic representation of PDGF receptor expression, scar size, myofibroblasts and vessel morphology in scars from different experimental groups.

Different PDGFs bind with different affinity to the two PDGF receptors (PDGFRs). We therefore mapped PDGFR expression in the cardiac tissue. PDGFRα-expressing cells were visualized by GFP expression. The number and localization of PDGFRα positive cells in scars was compared to the neighboring, histologically normal, myocardium (Fig 4A–4F). In empty vector scars, the abundance of *Pdgfra* expressing cells was unchanged in comparison to the surrounding non-fibrotic myocardium. In contrast, all scars generated with PDGF expressing viruses displayed an increase in the number of PDGFRα positive cells. The distribution pattern of PDGFRα positive cells varied between the different PDGF scars, but the levels were highly consistent within each group, in spite of variations in scar size. PDGFRα positive cells appeared homogeneously distributed throughout the scar tissue in some of the mice, but were locally concentrated to in others. We therefore assessed the density of PDGFRα positive cells in central scar areas, around blood vessels and in the scar edges (Fig 4G–4I). The highest density of PDGFRα positive cells was observed in the PDGF-D scars. In PDGF-A_long_ scars, the highest levels of PDGFRα positive cells were found in the central scar area, whereas the scar periphery had densities of PDGFRα positive cells comparable to the surrounding normal myocardium. PDGF-B scars also showed focal concentration of PDGFRα positive cells, which occurred in dense clusters without a preferential central-peripheral or vascular association.

PDGFRβ expression was observed in close proximity to the blood vessels in all scars (Fig 4A–4F). In contrast to PDGFRα, no apparent differences in distribution were observed between the different groups. A drawing (Fig 4K) schematically illustrates the PDGFR distribution patterns in the different scars.

### Ligand-specific inflammatory responses

**Fig 5:**
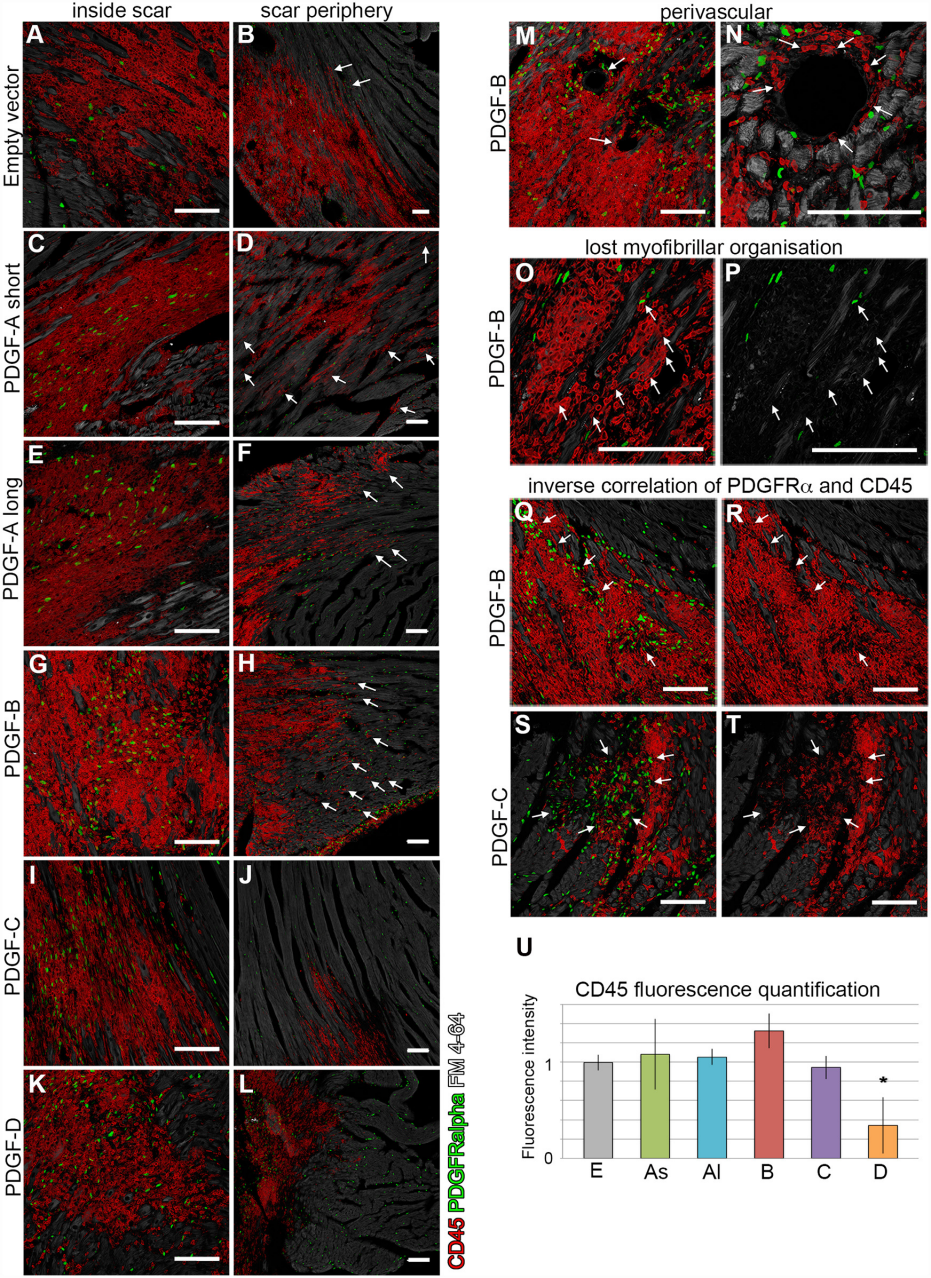
Inflammatory response in virus-induced scars. Expression of the pan-inflammatory marker CD45 (red), PDGFRα (green) and lipid membranes (grey) in center and periphery of scars. (Left column, A, C, E, G, I, K) Abundant expression of CD45 in scars of all viruses. (B, D, F, H, J, L) Arrows indicate inflammatory cells outside of the scar area. (M, N) Arrows mark inflammatory cells that accumulate around blood vessels in PDGF-B induced scars. (O, P) The myofibrillar organization is lost where inflammatory cells invade the myocardium in PDGF-B induced scars (arrows). The red channel has been removed in (P) to visualize absence of grey staining in the lost myofibrils. (Q-T) CD45 and PDGFRα are inversely expressed in PDGF-B and PDGF-C induced scars. The green channel has been removed in (R and T) to visualize the inverse correlation. (U) Quantification of cells expressing CD45 in the different experimental groups by fluorescence intensity measurements. All values are normalized to the empty vector control group, error bars show standard deviation and statistical significance is represented by asterisk (p<0.05). E = empty vector; As = PDGF-A_short_; Al = PDGF-A_long_; B = PDGF-B; C = PDGF-C; D = PDGF-D. Scale bars are 100μm.

Adenoviruses induce local inflammatory reactions [17,18]. The variations in scar sizes induced by the different PDGF viruses suggest PDGF ligand-specific modulation of inflammatory responses. We used the pan-inflammatory cell marker CD45 to investigate the presence and distribution of inflammatory cells in the scar tissue (Fig 5). Empty virus vector scars exhibited a prominent accumulation of homogeneously distributed CD45 positive cells (Fig 5A). The concentration of inflammatory cells in PDGF-A_short_, -A_long_ and -C scars was equal or lower than in empty vector controls (Fig 5C, 5E and 5I). In PDGF-D scars, the number of CD45 positive cells was markedly lower compared with all other scars (Fig 5K), a difference that was statistically significant also by fluorescence intensity (Fig 5U).

PDGF-B scars displayed a different pattern, in which CD45 positive cells appeared in high cell density clusters (Fig 5G). Massive accumulation of CD45 positive cells was observed around blood vessels (Fig 5M and 5N). In PDGF-B scar periphery with abundant CD45 cells, muscle fibers had lost normal myofibril organization (arrows in Fig 5O and 5P). In PDGF-B and -C scars, presence of CD45 positive cells correlated inversely with the presence of PDGFRα positive cells (arrows in Fig 5Q–5T).

In scars generated by the empty virus vector, PDGF-C or PDGF-D, CD45 positive cells were mostly confined to the scar area and did not distribute into surrounding intact muscle (Fig 5B, 5J and 5L). This contrasted with PDGF-A_short_, -A_long_ and -B scars, in which CD45 positive cells were also present in the interstitium between the muscle fibers outside of the scar borders (arrows in Fig 5D, 5F and 5H).

### Vascular morphology

**Fig 6:**
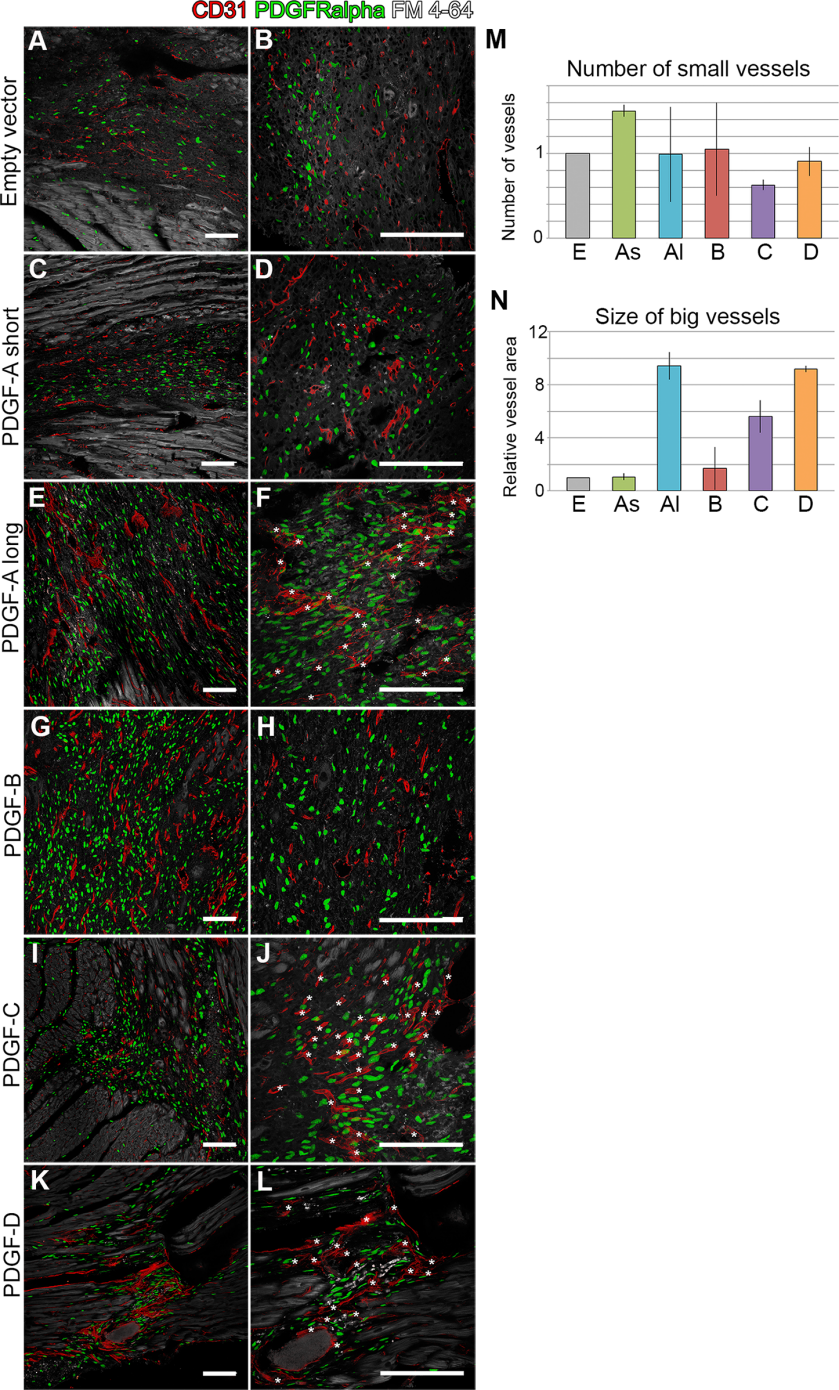
Angiogenic response in virus-induced scars. Endothelial CD31 expression (red) marks blood vessels, PDGFRα in green and lipid membranes in grey. (Left column, A, C, E, G, I, K) Overview, showing changes in vessel morphology and density in PDGF induced scars. (Right column, B, D, F, H, J, L) Asterisks mark big vessels with altered morphology. (M) Quantification of small vessels in the different experimental groups, normalized to the empty vector control. (N) Quantification of vessel size (area of cross-sectioned vessels) for the different experimental groups, normalized to the empty vector control. Analyses were performed on 3–4 mice per experimental group. E = empty vector; As = PDGF-A_short_; Al = PDGF-A_long_; B = PDGF-B; C = PDGF-C; D = PDGF-D. Scale bars are 100 μm.

Blood vessel morphology was assessed by CD31 labeling of endothelial cells (Fig 6A–6L). Empty vector scars were characterized by abundant small vessels of regular shape (Fig 6A and 6B). A similar pattern was seen in the PDGF-A_short_ scars (Fig 6C and 6D). The vessel pattern in the PDGF-B scars was reminiscent of that in the normal myocardium, with an even distribution of both small and large vessels (Fig 6G and 6H).

The vessel morphology was markedly different in scars generated by PDGF-A_long_, -C and -D expressing viruses, where abnormal clusters of large caliber vessels appeared (asterisks in Fig 6F, 6J and 6L). The vessel profiles were particularly prominent in the PDGF-D scars (Fig 6K and 6L). To quantify the changes in vessel morphology, we counted and measured small and large diameter vessel cross-section profiles in the scars (Fig 6M and 6N). Large diameter vessel cross-sections were present almost exclusively in scars induced by PDGF-A_long_, -C and–D viruses.

### Markers for cell repair, degeneration and proliferation

**Fig 7:**
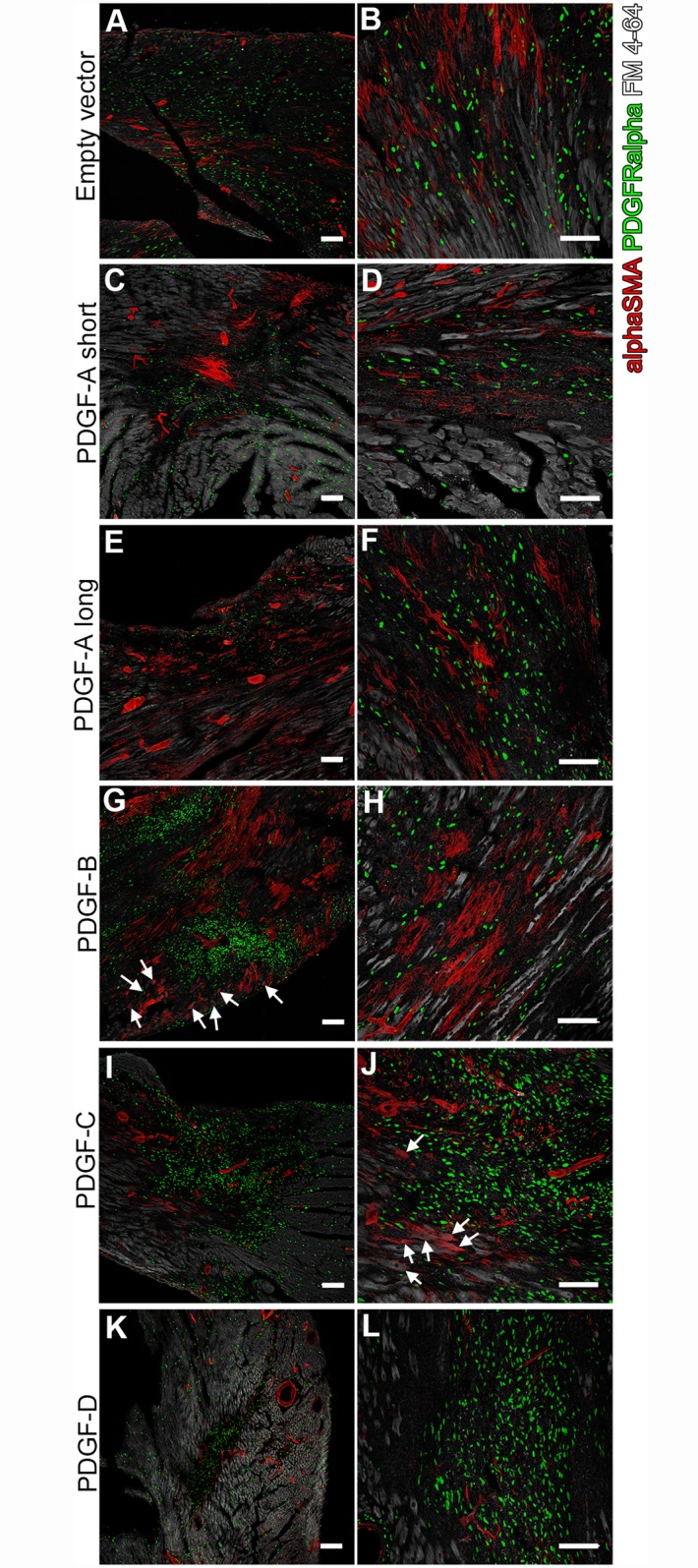
Myofibroblasts in the inflammatory response to viruses. Alpha-SMA (red) marks myofibroblasts and mural cells in the scars, PDGFRα is in green and lipid membranes in grey. Arrows in (G) and (J) point at α-SMA positive cardiomyocytes outside of scars induced by PDGF-B and PDGF-C viruses. Scale bars are 100 μm.

Inflammation is believed to lead to activation and transformation of fibroblasts/fibrocytes into alpha-smooth muscle actin (Acta2/α-SMA)-positive myofibroblasts. In addition, α-SMA is a common marker for differentiating cardiomyocytes during injury and remodeling [19,20]. In empty vector scars, α-SMA was expressed in large patches of myocardial-like fibers that were not vessel-associated (Fig 7A and 7B). A similar pattern was seen in PDGF-A_long_ and PDGF-B scars (Fig 7E–7H). In PDGF-A_long_, -A_short_ and to some extent also in PDGF-C scars, small clusters of intensely stained α-SMA positive cells were observed (Fig 7C–7F, 7I and 7J). Apart from mural cells, no α-SMA was observed in the PDGF-D virus-induced scars (Fig 7K and 7L). In scars induced by PDGF-B and -C viruses, α-SMA positive cardiomyocytes were also seen in normal cardiac tissue near the scar borders (arrows in Fig 7G and 7J).

**Fig 8:**
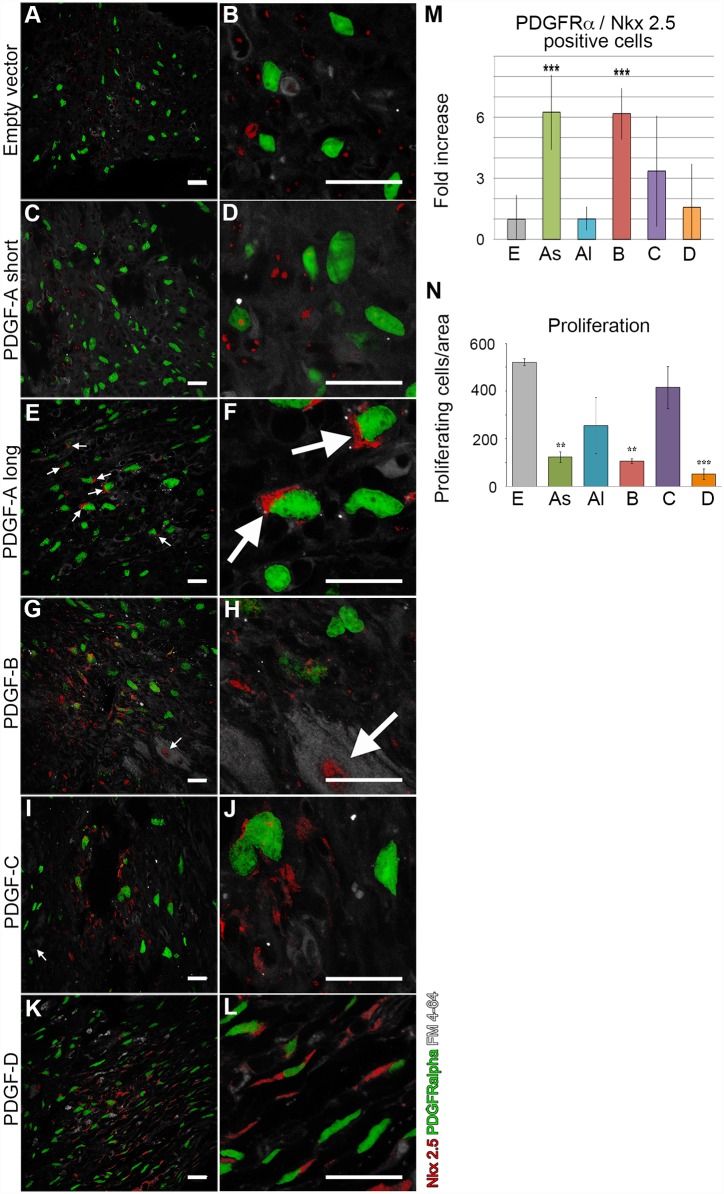
Co-expression of the myocardial precursor protein Nkx2.5 and PDGFRα. Nkx2.5 (red), PDGFRα (green) and lipid membranes (grey) in virus-induced scars. (A, B) No co-expression in scars induced by empty vector control viruses. (C, D) High number of co-expressing cells after PDGF-A_short_ viruses. (E, F) Perinuclear localization of Nkx2.5 in scar from PDGF-A_long_ virus. (G, H) Arrow indicates Nkx2.5 positive cardiomyocyte surrounding the scar region in a PDGF-B induced scar. (I, J) Scar from PDGF-C virus. (K, L) Scar from PDGF-D virus. (M) Quantification of cells coexpressing Nkx2.5 and PDGFRα in the different experimental groups. All values are normalized to the empty vector control, error bars show standard deviations and asterisks indicate the statistical significance (p<0.001). (N) Quantification of proliferative response to the viral injections in the different experimental groups. E = empty vector; As = PDGF-A_short_; Al = PDGF-A_long_; B = PDGF-B; C = PDGF-C; D = PDGF-D. Scale bar 20 μm.

Nkx2.5 expression is specific for the heart [21] and is often used to label myocardial precursor cells and remodeling myocardium. We analyzed Nkx2.5 to identify a potential overlap with PDGFRα8). The empty vector scars contained Nkx2.5 positive cells, but these did not co-localize with PDGFRα (Fig 8A and 8B). This contrasted with the PDGF-virus induced scars which all contained Nkx2.5/ PDGFRα double positive cells (Fig 8M). The highest amounts of double positive cells were seen in PDGF-A_short_ and -B scars, where over 30% of the PDGFRα positive cells expressed Nkx2.5. These results should be interpreted with some caution due to the different subcellular distribution of the two reporters (cytoplasm for Nkx2.5 immunostaining and nuclear for *Pdgfra*-H2BGFP; arrows in Fig 8E and 8F). In a few cases of the PDGF-B and -C scars, positive Nkx2.5 nuclei were visible in cardiomyocytes surrounding the scar tissue as well (arrows in Fig 8H).

The different scar sizes generated by different PDGF viruses suggest possible differences in cell proliferation. The overall cell proliferation in the scars was estimated using Ki67 antibodies, which detect an antigen present in the nucleus during all active phases of the cell cycle (Fig 8N). Somewhat surprisingly, the highest proliferative activity was observed in the empty vector control and PDGF-C scars. Compared with empty vector, PDGF-A_long_ scars showed a 50% lower density of Ki67 positive cells, and in PDGF-A_short_, -B, and -D scars, the proportion of Ki67 positive cells was only 10–20% of that in empty vector scars.

## Discussion

Earlier gain-of-function studies have consistently implicated PDGFs/PDGFRs as pathogenic drivers in tissue fibrosis of different organs (reviewed in [1]). In the heart, transgenic expression of PDGF-C [11], PDGF-D [12], PDGF-A and PDGF-B (Gallini et al, submitted) all induced cardiac fibrosis to different extent. PDGF-A and-C (that signal through PDGFRα) induced widespread fibrosis, while PDGF-B and -D (that signal primarily through PDGFRβ) generated local blood vessel-associated fibrotic reactions. The mentioned results were elicited through transgenic PDGF overexpression from the cardiac myosin heavy chain promoter, which is active already during cardiac development. To assess the role of PDGFs during cardiac fibrosis in adult pathology, we induced PDGF expression in the adult healthy heart in conjunction with adenovirus-induced inflammation. A similar approach has previously been applied in heterotypic heart transplants in rats; in these studies, adenovirus-mediated transfer of PDGF-A, -C, and -D accelerated cardiac fibrosis and chronic rejection, implicating PDGFs as drivers of transplantation-associated cardiac fibrosis [22]. The results reported herein suggest that the state of inflammation exerts a strong influence on the PDGF-isoform-specific cardiac effects.

We aimed to compare the fibrogenic propensities of the different PDGFs in the adult heart and to identify a putative fibrogenic target cell for the PDGF ligands. We hypothesized that PDGFRα positive cells are key players in the fibrotic reaction. This was based on observed *Pdgfra* expression patterns during heart development, as well as the phenotypes of α-MHC-PDGF transgenic mice (Gallini et al., submitted). We also expected results matching the known ligand-receptor interactions known to occur *in vivo* during development (PDGF-A and -C via PDGFRα and PDGF-B via PDGFRβ; [1]). Indeed, we found that PDGFRα positive cells were involved in the fibrogenic response, because the numbers of PDGFRα positive cells were increased in scars generated by all PDGF viruses, but not by empty vector virus.

The importance of PDGFRα was further implicated by the differences between PDGF-A_long_ and PDGF-A_short_ scars. The PDGF-A_short_ isoform generated small scars with homogeneous distribution of PDGFRα positive cells, whereas the PDGF-A_long_ isoform induced large scars with PDGFRα-positive cells concentrated at the scar center (i.e. close to the virus injection site). The PDGF-A_short_ scars also contained more PDGFRα/Nkx2.5 double positive cells than the larger scars of PDGF-A_long_. We do not know if the occurrence of scar size and double positive cells are causally related, but it is likely that the scar size reflects bioavailability and diffusion of PDGF-A in the tissue. PDGF-A_short_ is thought to be freely diffusible in contrast to PDGF-A_long_ that binds extracellular matrix and accumulates around the producing cells. It is not know if the high accumulation of PDGF-A_long_ stimulates Nkx2.5 expression.

The high density of PDGFRα-positive cells in PDGF-D scars was unexpected. PDGF-D has been reported to be a weak agonist for PDGFRα but PDGF ligand-receptor interactions may differ during development and pathology. PDGF-B, which binds both PDGFRs, had a markedly different effect than PDGF-D. It seems plausible that the contrasting effects of PDGF-B and PDGF-D viruses relate to the known differences in PDGFR specificities; *in vitro*, PDGF-D only binds to PDGFRβ [23], whereas PDGF-B binds PDGFRα, PDGFRβ and PDGFR heterodimers [24,25]. During development, PDGF-B acts mainly via PDGFRβ (knockouts for the two are indistinguishable) [26], but the receptor interactions in inflammation and fibrosis are less well understood. It is also possible that the PDGF-D virus effects are caused indirectly. Perhaps PDGF-D modulates extracellular matrix deposition, eg. by shifting the balance between MMPs/MMP inhibitors [27], or by reducing tissue edema, thereby generating small scars with high cell density.

Somewhat surprisingly, the number of proliferating cells in the scars at 2 weeks post virus injection did not positively correlate with scar size. We cannot exclude earlier effects of proliferation since the cellular composition of the scars was already quite complex two weeks after virus injection. No significant effects on ejection fractions were observed in the PDGF adenovirus groups, but it should be kept in mind that not only the scar size affects heart functionality. Cellular composition of the scar is important, for example, a large injured area might contain more functional cardiomyocytes compared to a small fibrotic scar. Also, a small scar that reaches from the epicardium to the endocardium might deteriorate cardiac function more than a large, centrally located, scar. We have previously shown that the ejection fraction is less reduced in adenoviral scars than in diffuse fibrosis that causes stiffness of the total left ventricle [18].

The questions if PDGFs modify inflammatory responses directly or indirectly, and conversely if inflammatory mediators affect PDGF activity, remain open. Fibroblasts are increasingly recognized as active cells contributing to inflammatory processes (reviewed by [28]). Differences in scar size might reflect differences in cytokine release, which could be specific to the different PDGF viruses. Regardless, our data suggest the existence of a PDGF isoform-specific modulation of inflammatory responses. Inflammation (here measured by the intensity of CD45 expression) varied extensively between scars. PDGF-B and–D, the two PDGFRβ-binding ligands, generated scars in which the inflammatory response was altered in opposite ways: The PDGF-B virus generated large scars with increased ventricle wall thickness and extensive inflammation, whereas PDGF-D scars were small, compact and less inflammatory. It is not unlikely that inflammation itself may influence the responses to PDGF-B and PDGF-D differently, perhaps through differential proteolytic activation of latent PDGF-D, or processing of the PDGF-B C-terminus, which might change its bioavaiability.

The extensive inflammation observed in PDGF-B scars is in agreement with data reported by others. PDGF/PDGFR signaling has been linked to the inflammatory state in desoxycorticosterone-induced myocardial fibrosis in rats [29]. A recent study suggested that pericytes and mesenchymal cells may express immune response genes following PDGFRβ signaling, leading to enhanced recruitment of leukocytes [30]. Pericytes have been suggested to play an active role in innate leukocyte guidance [31]. A direct implication of PDGFRβ signaling in local leucocyte accumulation, VSMC dedifferentiation and inflammation has also been demonstrated in a model of atherosclerosis [32]. Our results suggest that these PDGF guided inflammatory reactions might be PDGF-B isoform-specific.

The presence and distribution of α SMA positive myofibroblasts in the adenovirus-induced scars varied between the different PDGF viruses. Interestingly, the PDGF-D scars were essentially devoid of αSMA positive myofibroblasts. PDGF signaling linked to cell fate determination has been observed in αSMA positive mural cells during atherosclerosis [32]. Normally, the presence of myofibroblasts promotes tissue contraction and wound healing, but they may also give rise to an excessive accumulation of extracellular matrix and the formation of a fibrotic scar [33]. Thus, myofibroblasts may have both positive and negative functions in tissue repair and recovery. In myocardial fibrosis, PDGF/PDGFR signaling has been suggested to stimulate fibroblasts to proliferate and transform into collagen-deposing myofibroblasts. When considering PDGF/PDGFR signaling as a putative therapeutic target in tissue fibrosis, it should be remembered that a specific signaling pathway might produce beneficial responses in certain contexts and pathogenic responses in others. Also, the different signaling pathways induced by PDGFRα and PDGFRβ may suggest the need for receptor subtype-specific inhibitors in order to achieve beneficial effects. For example, responses to myocardial infarction were differentially modulated by antibodies against PDGFRβ (inhibition of vascular maturation) or PDGFRα (decreased collagen deposition) [34].

Our results using PDGF adenoviruses in the adult mouse heart challenge our current views on PDGF biology. Our data provide evidence for PDGF isoform-specific effects on the scar composition during inflammation. We would not have predicted all of the observed effects, nor considered some of the scenarios discussed above, based on prior knowledge of PDGF ligand-receptor specificities. PDGF signaling has been studied extensively *in vitro*, since the first PDGFs were identified during the early 70s. Many questions remain concerning PDGF signaling *in vivo*, including the existence of putative co-receptors, active ligand- and/or receptor heterodimers, and the regulation of ligand bioavailability. These are all largely unexplored areas of putatively critical importance for physiological and pathological responses. At present, we do not understand how binding of different PDGF isoforms to the same receptor may generate different readouts.

In conclusion, our study highlights ligand/receptor-specific effects of different PDGF isoforms in conjunction with inflammation in the heart. PDGF-BB is the commonly applied PDGFR agonist in various types of experiments, and its effects are often considered representative for all the PDGF isoforms. Our results instead suggest that PDGF-induced responses may differ profoundly between PDGF ligands and whether the responses are elicited in a developmental or inflammatory context.
